# Incident generalized anxiety and common mental disorders in rheumatoid arthritis: a 12-year case-cohort analysis from ELSA-Brasil

**DOI:** 10.1007/s00296-026-06339-2

**Published:** 2026-11-05

**Authors:** Patrícia Fonseca Estrada, Marcos Rafael Nogueira Cavalcante, Itamar de Souza Santos, Andre Russowsky Brunoni, Vandrize Meneghini, William Rodrigues Tebar, Paulo Andrade Lotufo, Alessandra de Carvalho Goulart, Isabela Judith Martins Bensenor

**Affiliations:** 1 Clinical and Epidemiological Research Center, University Hospital, University of São Paulo, Av. Lineu Prestes, 2565, 3rd floor, São Paulo, SP Brazil; 2 School of Public Health, University of São Paulo, São Paulo, Brazil

**Keywords:** Arthritis, Rheumatoid, Depressive disorder, Generalized anxiety disorder, Mental disorders, Cohort studies

## Abstract

Rheumatoid arthritis (RA) is associated with mental health disorders, but longitudinal evidence remains limited. To examine the association of RA with depressive episode, generalized anxiety disorder (GAD), and common mental disorders (CMD) in the Brazilian Longitudinal Study of Adult Health (ELSA-Brasil). This case-cohort analysis included 1,795 participants: 252 in the RA case group and 1,543 in a randomly selected reference sample. For each incident outcome, participants with the condition at baseline or without follow-up information were excluded. Outcomes were assessed over 12 years of follow-up. Logistic regression estimated odds ratios (ORs) and 95% confidence intervals (CIs), adjusted for sociodemographic and clinical covariates. At baseline, depressive episode was present in 19 (7.5%) participants in the RA group and 60 (3.9%) in the reference sample, GAD in 45 (18.1%) and 186 (12.2%), and CMD in 92 (36.5%) and 411 (26.6%), respectively. Fully adjusted ORs for the association of RA with depressive episode, GAD, and CMD were 1.52 (95% CI 0.85–2.74), 1.30 (0.89–1.90), and 1.41 (1.04–1.90), respectively. During follow-up, depressive episode occurred in 24 (14.4%) participants in the RA group and 94 (9.4%) in the reference sample, GAD in 37 (25.2%) and 120 (13.3%), and CMD in 43 (36.4%) and 164 (20.9%), respectively. Corresponding fully adjusted ORs were 1.41 (0.84–2.36), 2.04 (1.30–3.18), and 2.06 (1.33–3.18), respectively. RA was associated with baseline CMD and incident GAD and CMD, but not with baseline depressive episode, baseline GAD, or incident depressive episode after adjustment.

## Introduction

Rheumatoid arthritis (RA) is a chronic immune-mediated systemic disease characterized by persistent synovial inflammation, progressive joint damage, and potential impairment of mobility, physical function, and daily activities [1]. Although articular involvement is its principal clinical manifestation, RA may also affect multiple organs and tissues through extra-articular manifestations [2]. Estimates from the Global Burden of Disease 2021 indicated approximately 17.92 million prevalent cases of RA worldwide in 2021, with substantial variation according to age, sex, geographic region, and sociodemographic context [3]. This heterogeneity reinforces the relevance of investigating RA-related outcomes in populations with different demographic and epidemiological characteristics [3].

Mental health disorders are frequently reported among individuals with RA, particularly depressive and anxiety disorders [4]. A meta-analysis restricted to studies using semi-structured diagnostic interviews estimated current prevalences of 17.9% for depressive disorders and 13.5% for anxiety disorders, with corresponding lifetime estimates of 32.4% and 22.2% [4]. However, the reported estimates varied considerably across studies, and men, non-White populations, and individuals with young adult-onset RA were underrepresented [4]. These findings indicate that the estimated burden of psychiatric morbidity in RA is influenced by outcome definition, assessment method, study design, and population composition.

The clinical relevance of depressive and anxiety disorders in RA extends beyond their frequency. Studies have examined their associations with treatment response, physical disability, patient-reported outcomes, and perceived health status [5–7]. In a pooled analysis of five randomized controlled trials involving patients with moderate-to-severe RA, comorbid depression was associated with a lower likelihood of remission, whereas anxiety was not independently associated with remission after adjustment [6]. Cross-sectional studies have also reported associations of depressive and anxiety symptoms with patient global assessment, functional impairment, and poor self-rated health, although findings for composite measures of RA disease activity have been inconsistent [5, 7]. These differences support the evaluation of depressive and anxiety disorders as related but distinct psychiatric outcomes in RA research.

Despite the documented frequency and clinical relevance of psychiatric morbidity in RA, important gaps remain. Much of the available evidence is based on cross-sectional studies, which cannot distinguish mental health disorders already present at the time of assessment from new outcomes occurring during follow-up [5, 7–11]. Longitudinal investigations are therefore necessary to determine whether RA is associated with the subsequent development of psychiatric disorders [12–14]. In a Canadian population-based matched cohort study, individuals with RA had higher incidence and prevalence of depressive and anxiety disorders than matched individuals without RA [13]. A systematic review and meta-analysis of cohort studies also identified a bidirectional association between RA and depression, including evidence of an increased subsequent risk of depression among individuals with RA [12]. However, few studies have combined baseline and incident analyses using standardized psychiatric assessments within the same cohort. Evidence is particularly limited for common mental disorders (CMD), a broader construct that captures clinically relevant non-psychotic psychiatric morbidity beyond diagnosis-specific categories [14].

Using data from ELSA-Brasil, the present study examined the association of RA case group membership with baseline and incident depressive episode, generalized anxiety disorder (GAD), and CMD. Baseline analyses assessed psychiatric morbidity present at study entry, whereas longitudinal analyses evaluated cumulative incident outcomes using outcome-specific assessments available through the 12-year follow-up visit.

## Materials and methods

### Study design and population

ELSA-Brasil is a prospective multicentre cohort comprising 15,105 civil servants aged 35 to 74 years at baseline, recruited from six Brazilian cities between 2008 and 2010. The cohort was designed to investigate the incidence and determinants of cardiovascular diseases, diabetes, and other chronic conditions [15]. The 2008–2010 period corresponds to the baseline examination. For the present study, baseline data and repeated mental health assessments collected during subsequent ELSA-Brasil follow-up visits through the 12-year examination were used. The outcome-specific assessment schedule is described below.

The present study was conducted within the Chronic Inflammatory Diseases ancillary project, which adopted a case-cohort sampling design including 3,965 ELSA-Brasil participants [16]. Participants included in the ancillary study underwent rheumatoid factor and anti-CCP measurements. At ELSA-Brasil inception, the Aleatory Cohort Sample (ACS) was established as a random subsample comprising approximately 10% of the baseline cohort (1,543 of 15,105 participants), with the purpose of representing the underlying cohort and serving as a fixed reference sample for ancillary studies according to a case-cohort design. Selection into the ACS was independent of RA status and of the mental health outcomes evaluated in the present study. The remaining 252 participants who met the RA definition constituted the RA case group. Participants from this case-cohort source population, using the same RA ascertainment strategy and ACS reference framework, have also been evaluated in previous ELSA-Brasil studies of coronary and carotid subclinical atherosclerosis [17, 18]. Participants in the ACS underwent the same standardized cohort assessments as other ELSA-Brasil participants, with additional biological samples collected and stored for ancillary investigations [16]. In the present study, part of these stored biological samples was used to determine rheumatoid factor and anti-CCP levels.

The ELSA-Brasil protocol was approved by the Research Ethics Committees of all six participating centers: the Institute of Collective Health, Federal University of Bahia (UFBA; approval no. 027/06; May 26, 2006); Oswaldo Cruz Foundation (FIOCRUZ; approval no. 343/06; September 18, 2006); University Hospital, University of São Paulo (HU-USP; approval no. 669/06; May 19, 2006); Federal University of Minas Gerais (UFMG; approval no. 186/06; June 28, 2006); Health Sciences Center, Federal University of Espírito Santo (UFES; approval no. 041/06; May 31, 2006); and Hospital de Clínicas de Porto Alegre, Federal University of Rio Grande do Sul (HCPA-UFRGS; approval no. 194/06; May 15, 2006). The present research project was additionally approved by the Research Ethics Committee of the University Hospital, University of São Paulo (Comitê de Ética em Pesquisa do Hospital Universitário da Universidade de São Paulo, HU-USP; CAAE 78744824.0.0000.0076; approval no. 6,760,809; April 12, 2024). All participants provided written informed consent before enrollment in ELSA-Brasil. All procedures were conducted in accordance with the Declaration of Helsinki and its subsequent amendments [15].

This study was reported in accordance with the Strengthening the Reporting of Observational Studies in Epidemiology (STROBE) Statement for cohort studies [19].

### Definition of rheumatoid arthritis

RA classification was based on information collected during ELSA-Brasil examinations, including self-reported physician diagnosis, use of disease-modifying antirheumatic drugs (DMARDs), and rheumatoid factor (RF) and anti-cyclic citrullinated peptide antibodies (anti-CCP) as serological markers. Participants were asked about previous physician diagnoses of RA. Medication use was assessed based on drugs taken during the 14 days preceding the study visit. Serum samples were tested for RF and anti-CCP, as previously described [16].

Participants were classified as having RA when at least two of the following three criteria were present: self-reported physician diagnosis of RA, DMARD use during the previous 14 days, and seropositivity for RF or anti-CCP. This operational definition was used for epidemiological case ascertainment in the absence of systematic rheumatologist assessment and is consistent with approaches previously applied in large population-based cohorts [20–22].

### Mental health outcomes

Depressive episode, GAD, and CMD were assessed using the Clinical Interview Schedule-Revised (CIS-R), administered during ELSA-Brasil examinations by trained lay interviewers according to standardized cohort procedures [23, 24]. The CIS-R is a structured interview developed to assess non-psychotic psychiatric morbidity in community and primary care settings and permits the classification of psychiatric disorders using ICD-10-based diagnostic algorithms [25, 26]. For use in ELSA-Brasil, the instrument was translated and culturally adapted into Brazilian Portuguese [25].

Depressive episode and GAD were defined using CIS-R-derived ICD-10 diagnostic algorithms. Depressive episode included mild, moderate, and severe episodes, with or without somatic symptoms. GAD was defined according to the corresponding diagnostic algorithm [25]. CMD was defined as a CIS-R total score of 12 or higher. The total score ranges from 0 to 57, with higher scores indicating greater non-psychotic psychiatric morbidity. CMD represents a broader measure of clinically relevant psychiatric morbidity and is not equivalent to a specific ICD-10 diagnosis [25].

Baseline prevalence and cumulative incidence were defined separately for each outcome. Baseline prevalence analyses included participants with available data for the corresponding outcome. For incidence analyses, participants with the respective outcome at baseline were excluded. Depressive episode was assessed at baseline and at the 4-year, 8-year, and 12-year follow-up visits. GAD and CMD were assessed at baseline and at the 8-year and 12-year follow-up visits and were not assessed at the 4-year visit.

Incident depressive episode, GAD, and CMD were defined as the occurrence of the corresponding outcome at one or more available follow-up visits among participants without that outcome at baseline. Each incident outcome was analyzed as a cumulative binary indicator. Because the availability of CIS-R-derived measures differed across study waves and outcomes, separate analytical samples were defined for each outcome.

### Study participants

**Fig. 1:**
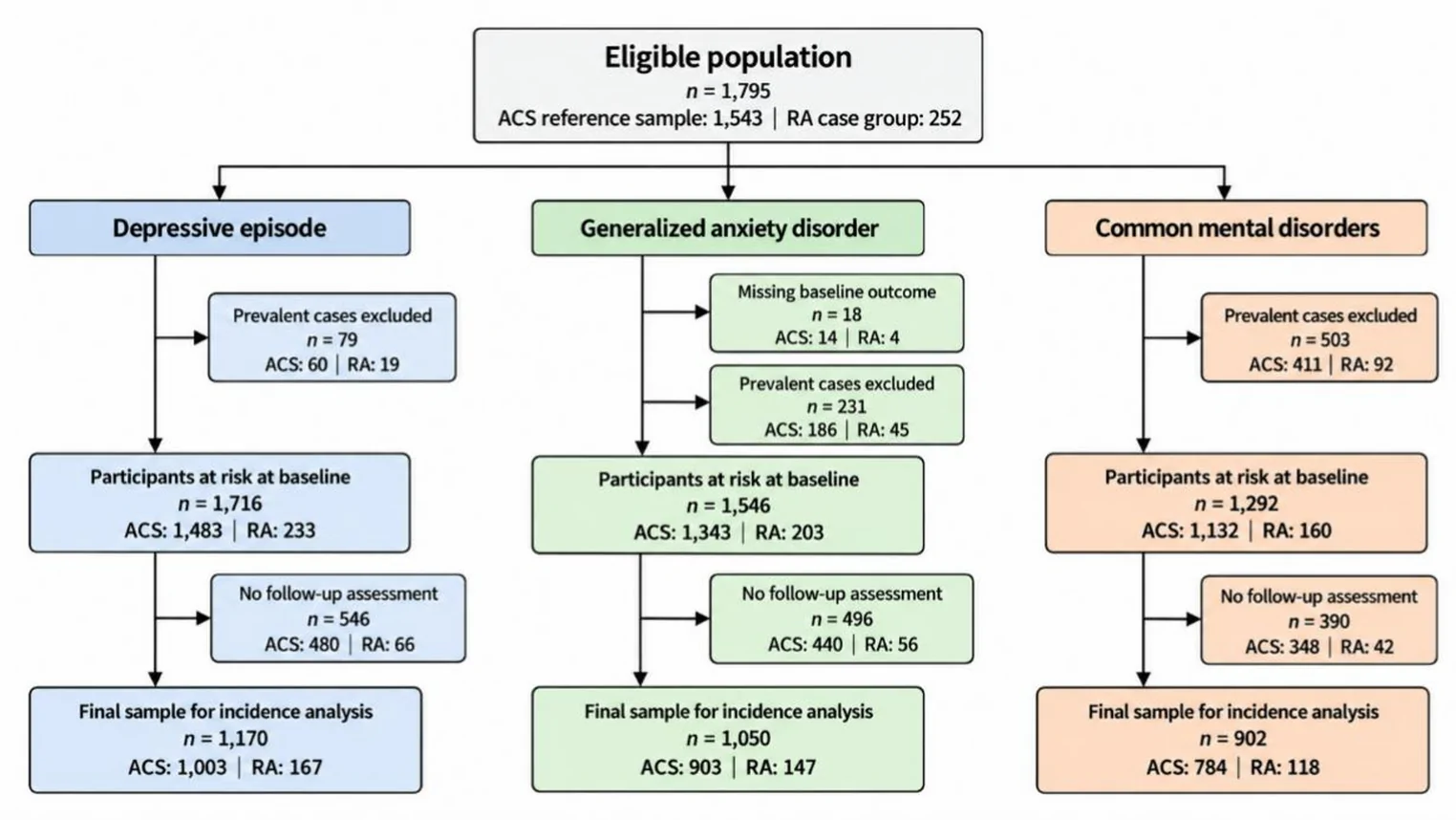
Flowchart of participant selection for the analyses of baseline and incident mental health outcomes in ELSA-Brasil. The eligible population comprised 1,795 participants, including 252 in the RA case group and 1,543 in the ACS reference sample. The ACS was selected independently of RA status; 27 ACS participants also met the operational RA definition and remained in the ACS. Outcome-specific exclusions were applied separately for each mental health outcome. *ACS* aleatory cohort sample; *CMD* common mental disorder; *GAD* generalized anxiety disorder; *RA* rheumatoid arthritis

The participant selection process is summarized in Fig. 1. The eligible study population comprised 1,795 participants, including 252 in the RA case group and 1,543 in the ACS reference sample.

Baseline analyses included participants with available information for each mental health outcome. Separate analytical samples were defined for the incidence analyses. For each outcome, participants with missing baseline information, when applicable, those with the corresponding outcome at baseline, and those without follow-up information for that outcome were excluded.

For depressive episode, 79 participants with the outcome at baseline and 546 without follow-up information were excluded, resulting in an analytical sample of 1,170 participants, including 167 in the RA case group and 1,003 in the ACS reference sample.

For GAD, 18 participants with missing baseline information, 231 with GAD at baseline, and 496 without follow-up information were excluded. The final analytical sample comprised 1,050 participants, including 147 in the RA case group and 903 in the ACS reference sample.

For CMD, 503 participants with the outcome at baseline and 390 without follow-up information were excluded, resulting in an analytical sample of 902 participants, including 118 in the RA case group and 784 in the ACS reference sample.

### Baseline covariates and descriptive variables

Sociodemographic and health-related data were collected at baseline using structured questionnaires and standardized procedures [15, 27]. Covariates included in the adjusted models were age, sex, race/ethnicity, educational attainment, and baseline antidepressant use. Age was analyzed as a continuous variable, and sex was categorized as female or male. Race/ethnicity was self-reported according to the categories used by the Brazilian national census: White, Black, Mixed, Asian, and Indigenous. Educational attainment was categorized as less than high school, high school or some college, and college graduate or higher [27]. Baseline antidepressant use was defined as current use at the baseline examination. Participants reported prescription and nonprescription medications, including continuous and non-continuous medications used during the previous two weeks, and were instructed to bring medication packages and prescriptions to the study clinic [23, 27, 28].

Additional variables were included for descriptive purposes only. Monthly household income was categorized according to predefined ELSA-Brasil categories and presented as approximate United States dollar equivalents: < $1,245, $1,245 to $3,319, and ≥ $3,320. These values were calculated using an approximate exchange rate of US$1 to R$2 during the baseline data collection period and were used only for descriptive presentation [27].

Marital status was categorized as married or partnered versus separated, divorced, single, or widowed. Smoking status was categorized as never, former, or current smoking, and alcohol consumption as never, former, or current use. Obesity was defined as body mass index ≥ 30 kg/m². Self-rated health was categorized as very good, good, fair, poor, or very poor [27].

### Statistical analysis

Baseline characteristics were described separately for the RA case group and the ACS reference sample. Categorical variables were presented as frequencies and percentages and compared using Pearson’s chi-square test. Age was presented as mean and standard deviation and compared using Student’s t-test.

Baseline and cumulative incidence analyses were conducted separately for depressive episode, generalized anxiety disorder, and common mental disorders. Baseline analyses included participants with available information for the corresponding outcome. For incidence analyses, participants with the outcome at baseline and those without follow-up information for that outcome were excluded. Incident outcomes were defined as the occurrence of the corresponding outcome at any available follow-up assessment.

Crude cumulative incidence was calculated as the proportion of participants who developed the corresponding outcome during follow-up among those without that outcome at baseline, separately for the RA case group and the ACS reference sample. Because the timing and number of follow-up assessments differed across outcomes, cumulative incidence estimates were interpreted within each outcome.

Logistic regression was used to estimate odds ratios (ORs) and 95% confidence intervals (CIs) for the association of RA case group membership with baseline and incident mental health outcomes, using the ACS reference sample as the reference group. Logistic regression was selected because exact dates of outcome onset were unavailable and mental health outcomes were assessed only at scheduled study visits.

For each outcome, a univariable logistic regression model was first fitted with RA case group membership as the sole predictor. Multivariable logistic regression models were then fitted sequentially. Model 1 was adjusted for age and sex; Model 2 was additionally adjusted for race/ethnicity and educational attainment; and Model 3 was further adjusted for baseline antidepressant use. Age was modeled as a continuous variable; sex, race/ethnicity, and educational attainment were modeled as categorical variables, and baseline antidepressant use as a binary variable. All regression analyses used complete-case data, and participants with missing information for any variable included in a given model were excluded from that model.

No sampling weights or other design-based weighting methods were applied. The ACS was retained as the predefined randomly selected cohort reference sample; selection into the ACS was independent of RA status. Analyses were performed using R software, version 4.6.1.

## Results

The flow of participants into each outcome-specific longitudinal analysis is shown in Fig. 1. In the eligible study population, participants in the RA case group were older than those in the ACS reference sample (mean age, 54.8 vs. 52.2 years; *p* < 0.001) and were more frequently women (75.8% vs. 54.1%; *p* < 0.001). The RA case group also had higher frequencies of obesity and baseline antidepressant use and a less favorable distribution of self-rated health. Other baseline characteristics are presented in Table 1.

At baseline, depressive episode, GAD, and CMD were more frequent in the RA case group than in the ACS reference sample (7.5% vs. 3.9%, 18.1% vs. 12.2%, and 36.5% vs. 26.6%, respectively). Crude logistic regression models showed statistically significant associations of RA case group membership with all three outcomes. In Model 3, adjusted for age, sex, race/ethnicity, educational attainment, and baseline antidepressant use, the association remained statistically significant for CMD (OR 1.41, 95% CI 1.04–1.90; *p* = 0.027). Associations with depressive episode (OR 1.52, 95% CI 0.85–2.74; *p* = 0.160) and GAD (OR 1.30, 95% CI 0.89–1.90; *p* = 0.176) were not statistically significant in the fully adjusted model (Table 2).

During follow-up, depressive episode occurred in 24 of 167 participants in the RA case group and 94 of 1,003 participants in the ACS reference sample, corresponding to cumulative incidences of 14.4% and 9.4%, respectively. GAD occurred in 37 of 147 participants in the RA case group and 120 of 903 participants in the ACS reference sample, corresponding to 25.2% and 13.3%, respectively. CMD occurred in 43 of 118 participants in the RA case group and 164 of 784 participants in the ACS reference sample, corresponding to 36.4% and 20.9%, respectively (Table 3).

RA case group membership was associated with incident depressive episode in the crude model (OR 1.62, 95% CI 1.00-2.63; *p* = 0.049), but the association was not statistically significant in the adjusted models. In Model 3, the OR was 1.41 (95% CI 0.84–2.36; *p* = 0.198). In contrast, the associations with incident GAD and CMD remained statistically significant across all models. In Model 3, RA case group membership was associated with higher odds of incident GAD (OR 2.04, 95% CI 1.30–3.18; *p* = 0.002) and incident CMD (OR 2.06, 95% CI 1.33–3.18; *p* = 0.001) compared with the ACS reference sample (Table 4).

## Discussion

In this longitudinal analysis of ELSA-Brasil, participants in the RA case group had approximately twice the odds of incident generalized anxiety disorder and common mental disorders compared with the ACS reference sample after adjustment for sociodemographic characteristics and baseline antidepressant use. In contrast, RA case group membership was not significantly associated with incident depressive episode in the adjusted models. At baseline, the association remained statistically significant only for CMD after full adjustment. These findings indicate that associations between RA and mental health outcomes may differ according to the psychiatric construct evaluated, with more consistent longitudinal associations for GAD and broader non-psychotic psychiatric morbidity than for depressive episode.

Cross-sectional studies have reported a substantial frequency of depressive and anxiety disorders among individuals with RA [5, 7–11]. A meta-analysis based on studies using diagnostic interviews also found elevated prevalences of these disorders, although estimates varied according to study design, diagnostic methods, and population characteristics [4]. Similarly, an analysis of the German National Cohort identified higher frequencies of physician-diagnosed depression and anxiety and clinically relevant symptoms among participants with inflammatory rheumatic diseases [29]. However, that study used self-reported rheumatic diagnoses and cross-sectional psychiatric assessments. The present study complements this evidence by using standardized CIS-R assessments and by evaluating both baseline and cumulative incident outcomes.

There are few prospective analyses of RA with mental disorders. These previous longitudinal studies generally support an association between RA and subsequent psychiatric morbidity. A Canadian population-based matched cohort study reported higher incidences of anxiety, depressive, and other psychiatric disorders among individuals with RA than among matched controls [13]. More recently, Giblon et al., in a population-based cohort with a mean follow-up exceeding 13 years, reported an increasing occurrence of anxiety among individuals with RA, whereas the risk of new depression diagnoses did not differ between RA and matched non-RA comparators. These findings are particularly relevant to the present results, in which RA case group membership was associated with incident GAD but not with incident depressive episode [14]. Dregan et al. also identified an increased risk of common mental disorders among individuals with chronic inflammatory conditions in a prospective primary care database study [30]. In addition, a systematic review and meta-analysis found evidence of a bidirectional association between RA and depression, including an increased subsequent risk of depression among individuals with RA [12]. The present study extends this literature by evaluating diagnosis-specific outcomes and CMD within the same longitudinal cohort and by defining separate at-risk samples for each outcome.

The association with incident GAD was one of the principal findings. Participants in the RA case group had approximately twice the odds of developing GAD during follow-up after adjustment. This result is consistent with population-based evidence indicating a higher frequency of anxiety disorders among individuals with RA [8, 13, 27] Most previous studies, however, have evaluated anxiety symptoms cross-sectionally [5, 7–9, 11, 29]. The present analysis provides longitudinal evidence based on a CIS-R-derived diagnostic algorithm rather than solely on symptom-screening scores. Nevertheless, the study did not assess the mechanisms underlying this association. Therefore, the findings cannot determine whether the increased occurrence of GAD was related to disease symptoms, functional limitations, treatment concerns, inflammatory activity, or other factors associated with RA.

CMD also showed a consistent pattern across the baseline and longitudinal analyses. RA case group membership was associated with CMD at baseline and with approximately twice the odds of incident CMD during follow-up. Unlike depressive episode and GAD, which were defined using ICD-10-based diagnostic algorithms, CMD was defined by a CIS-R total score of 12 or higher and captures a broader spectrum of clinically relevant non-psychotic psychiatric morbidity, including combinations of depressive, anxiety, somatic, and related symptoms [23, 25]. The association with CMD may therefore reflect psychiatric morbidity that is not fully captured by individual diagnosis-specific categories.

This finding differs from previous longitudinal studies and meta-analytic evidence reporting a higher incidence or risk of depression among individuals with RA [12, 13, 30]. Differences in outcome ascertainment may partly account for this variation. Previous studies have used administrative diagnoses, electronic health records, self-reported physician diagnoses, symptom-screening instruments, or combinations of these approaches. In the present study, depressive episode was defined using a CIS-R-derived ICD-10 algorithm that included mild, moderate, and severe episodes.

The occupational composition of ELSA-Brasil should be considered when interpreting these findings. Because the cohort comprises civil servants, participants may have had more stable employment and greater access to healthcare than the general population [15]. The cohort may also be subject to the healthy worker effect, whereby individuals who remain employed and participate in longitudinal assessments tend to have better overall health than those who are unable to remain in employment. This selection process may have resulted in greater representation of participants with milder or better-controlled RA. The observation of associations with incident GAD and CMD within this occupational cohort suggests that psychiatric morbidity was detectable even in a population potentially selected toward better health. However, RA activity and severity were not measured.

Baseline associations were less consistent than longitudinal associations. After adjustment, RA case group membership was associated with CMD but not with depressive episode or GAD at baseline. A recent population-based analysis from the Paracelsus 10,000 study found that participants with RA had approximately twice the adjusted odds of clinically relevant depressive symptoms compared with participants without RA [31]. However, that study used the BDI-II to assess depressive symptom burden and was cross-sectional, whereas the present study defined depressive episode using a CIS-R-derived ICD-10 diagnostic algorithm. Differences in psychiatric outcome definition and ascertainment may therefore contribute to the differing results. The association with baseline CMD in the present study may reflect the broader symptom range captured by this measure compared with the more restrictive diagnosis-specific algorithms. Cross-sectional associations may also be influenced by disease duration, previous RA treatment, adaptation to chronic illness, and psychiatric treatment before cohort entry. However, information on the timing of RA onset relative to psychiatric symptoms was unavailable, and the baseline analyses cannot establish temporal direction.

Depression, anxiety, and affective distress have been associated with treatment response, subsequent disease activity, and functional outcomes in RA [6, 32–34]. Psychiatric symptoms may affect treatment adherence, self-management, symptom perception, and healthcare use, while RA-related symptoms and limitations may contribute to psychological distress. Psychiatric comorbidity has also been associated with greater healthcare utilization among individuals with RA [35]. A recent study further showed that depressive and anxiety symptoms identified using standardized questionnaires were substantially more frequent among patients with RA than indicated by previously recorded clinical diagnoses, highlighting potential underrecognition of mental health morbidity in routine rheumatology care [36]. Although the present study was not designed to evaluate screening strategies, our longitudinal findings, particularly for incident GAD and CMD, reinforce the relevance of considering anxiety and broader psychiatric morbidity in addition to depression during the longitudinal assessment of individuals with RA.

This study has several strengths. It was conducted within ELSA-Brasil, a multicentre prospective cohort with standardized data collection. Mental health outcomes were assessed using the CIS-R, which provides standardized diagnostic algorithms and a measure of broader non-psychotic psychiatric morbidity. The study evaluated three distinct mental health outcomes and used outcome-specific longitudinal samples, excluding participants with the corresponding condition at baseline. The availability of repeated mental health assessments through the 12-year follow-up also permitted evaluation of cumulative incident outcomes.

**Table 1:** Baseline characteristics of the eligible study population by RA case group and ACS reference sample

Characteristic	RA (*n* = 252)	ACS (*n* = 1,543)	*p*-value
Age, years, mean (SD)	54.8 (9.3)	52.2 (8.9)	**< 0.001**
Female sex, n (%)	191 (75.8)	834 (54.1)	**< 0.001**
Race/ethnicity, n (%)			0.725
White	129 (51.8)	772 (50.4)	
Black	49 (19.7)	271 (17.7)	
Mixed	58 (23.3)	418 (27.3)	
Asian	9 (3.6)	50 (3.3)	
Indigenous	4 (1.6)	20 (1.3)	
Family income, n (%), USD			0.061
< $1,245	71 (28.3)	382 (24.8)	
$1,245 to $3,319	119 (47.4)	669 (43.5)	
≥ $3,320	61 (24.3)	487 (31.7)	
Education, n (%)			0.052
Less than high school	37 (14.7)	175 (11.3)	
High school/some college	97 (38.5)	523 (33.9)	
College or more	118 (46.8)	845 (54.8)	
Marital status, n (%)			**0.004**
Married/partnered	146 (57.9)	1,040 (67.4)	
Separated/divorced/single/widowed	106 (42.1)	503 (32.6)	
Smoking status, n (%)			0.449
Never	132 (52.4)	866 (56.2)	
Former	90 (35.7)	490 (31.8)	
Current	30 (11.9)	186 (12.1)	
Alcohol consumption, n (%)			**0.002**
Never	40 (15.9)	146 (9.5)	
Former	59 (23.5)	314 (20.4)	
Current	152 (60.6)	1,082 (70.2)	
Obesity (BMI ≥ 30 kg/m²), n (%)	80 (31.9)	314 (20.3)	**< 0.001**
Self-rated health, n (%)			**< 0.001**
Very good	40 (15.9)	432 (28.0)	
Good	129 (51.2)	834 (54.1)	
Fair	68 (27.0)	242 (15.7)	
Poor	11 (4.4)	28 (1.8)	
Very poor	4 (1.6)	7 (0.5)	
Depressive episode, n (%)	19 (7.5)	60 (3.9)	**0.014**
Generalized anxiety disorder, n (%)	45 (18.1)	186 (12.2)	**0.013**
Common mental disorders, n (%)	92 (36.5)	411 (26.6)	**0.002**
Baseline antidepressant use, n (%)	38 (15.3)	141 (9.2)	**0.004**

Values are mean (SD) or n (%). Percentages were calculated among participants with available data. P values were obtained using Student’s t test for age and Pearson’s chi-square test for categorical variables. Income categories are presented as approximate United States dollar equivalents. Missing data: race/ethnicity, RA *n* = 3 and ACS *n* = 12; household income, RA *n* = 1 and ACS *n* = 5; smoking status, ACS *n* = 1; alcohol consumption, RA *n* = 1 and ACS *n* = 1; obesity, RA *n* = 1; generalized anxiety disorder, RA *n* = 4 and ACS *n* = 14; antidepressant use, RA *n* = 3 and ACS *n* = 6

*ACS* Aleatory Cohort Sample; *BMI* body mass index; *CMD* common mental disorders; *RA* rheumatoid arthritis; *SD* standard deviation

**Table 2:** Association between rheumatoid arthritis and baseline mental health outcomes

Outcome	Model	OR (95% CI)	*p*-value
Depressive episode	Crude	2.02 (1.18–3.44)	**0.010**
	Model 1	1.87 (1.08–3.22)	**0.026**
	Model 2	1.68 (0.96–2.96)	0.072
	Model 3	1.52 (0.85–2.74)	0.160
Generalized anxiety disorder	Crude	1.60 (1.12–2.29)	**0.010**
	Model 1	1.41 (0.98–2.04)	0.065
	Model 2	1.34 (0.92–1.95)	0.122
	Model 3	1.30 (0.89–1.90)	0.176
Common mental disorders	Crude	1.58 (1.20–2.10)	**0.001**
	Model 1	1.49 (1.11–1.99)	**0.007**
	Model 2	1.43 (1.06–1.93)	**0.019**
	Model 3	1.41 (1.04–1.90)	**0.027**

ORs were estimated using logistic regression, with the ACS reference sample as the reference category. The crude model was unadjusted. Model 1 was adjusted for age and sex. Model 2 was additionally adjusted for race/ethnicity and educational attainment. Model 3 was further adjusted for baseline antidepressant use. Models were based on complete-case data

*ACS* Aleatory Cohort Sample; *CI* confidence interval; *CMD* common mental disorders; *GAD* generalized anxiety disorder; *OR* odds ratio; *RA* rheumatoid arthritis

**Table 3:** Cumulative incidence of mental health outcomes during follow-up in the RA case group and ACS reference sample

Outcome	RA		ACS	
	*N* at risk	Cases (%)	*N* at risk	Cases (%)
Depressive episode	167	24 (14.4%)	1,003	94 (9.4%)
Generalized anxiety disorder	147	37 (25.2%)	903	120 (13.3%)
Common mental disorder	118	43 (36.4%)	784	164 (20.9%)

Cumulative incidence was calculated as the proportion of participants with the corresponding outcome at any available follow-up assessment among those without that outcome at baseline and with follow-up information. Depressive episode was assessed at the 4-year, 8-year, and 12-year visits, whereas GAD and CMD were assessed at the 8-year and 12-year visits. Estimates should therefore be compared between the RA case group and ACS reference sample within each outcome, rather than directly across outcomes

*ACS* Aleatory Cohort Sample; *CMD* common mental disorders; *GAD* generalized anxiety disorder; *RA* rheumatoid arthritis

**Table 4:** Association of RA case group membership with cumulative incident mental health outcomes

Outcome	Model	OR (95% CI)	*p*-value
Depressive episode	Crude	1.62 (1.00-2.63)	**0.049**
	Model 1	1.61 (0.98–2.64)	0.060
	Model 2	1.40 (0.84–2.34)	0.195
	Model 3	1.41 (0.84–2.36)	0.198
Generalized anxiety disorder	Crude	2.19 (1.44–3.34)	**< 0.001**
	Model 1	2.13 (1.38–3.27)	**< 0.001**
	Model 2	2.00 (1.29–3.11)	**0.002**
	Model 3	2.04 (1.30–3.18)	**0.002**
Common mental disorders	Crude	2.17 (1.43–3.27)	**< 0.001**
	Model 1	2.09 (1.37–3.19)	**< 0.001**
	Model 2	2.01 (1.31–3.10)	**0.001**
	Model 3	2.06 (1.33–3.18)	**0.001**

ORs were estimated using logistic regression, with the ACS reference sample as the reference category. Incident outcomes were defined as occurrence at any available follow-up assessment among participants without the corresponding outcome at baseline. The crude model was unadjusted. Model 1 was adjusted for age and sex. Model 2 was additionally adjusted for race/ethnicity and educational attainment. Model 3 was further adjusted for baseline antidepressant use. Models were based on complete-case data

*ACS* Aleatory Cohort Sample; *CI* confidence interval; *CMD* common mental disorders; *GAD* generalized anxiety disorder; *OR* odds ratio; *RA* rheumatoid arthritis

Several limitations should be considered. RA was classified using an operational epidemiological algorithm rather than systematic rheumatologist assessment [21, 22]. Although the algorithm incorporated self-reported physician diagnosis, DMARD use, and autoantibody results, some degree of misclassification cannot be excluded. Information on RA duration, disease activity, pain, disability, and longitudinal treatment was unavailable. These factors are closely related to disease severity and mental health and may act as mediators, effect modifiers, or, depending on their temporal relationship, potential confounding factors. Their absence limited our ability to determine whether the observed associations with GAD and CMD were influenced by disease duration, inflammatory activity, symptom burden, functional impairment, or treatment exposure. Residual confounding and unmeasured mediation therefore cannot be excluded, and the magnitude of the observed associations should be interpreted accordingly. In addition, the healthy worker effect may have resulted in greater representation of participants with milder or better-controlled RA, potentially attenuating associations with mental health outcomes.

In conclusion, RA case group membership was associated with CMD at baseline and with higher odds of incident GAD and CMD during follow-up. No statistically significant association was observed with incident depressive episode after adjustment. These findings indicate that longitudinal psychiatric morbidity associated with RA may be more consistently detected for generalized anxiety and broader non-psychotic psychiatric morbidity than for depressive episode.
